# Brief Report: Isogenic Induced Pluripotent Stem Cell Lines From an Adult With Mosaic Down Syndrome Model Accelerated Neuronal Ageing and Neurodegeneration

**DOI:** 10.1002/stem.1968

**Published:** 2015-05-21

**Authors:** Aoife Murray, Audrey Letourneau, Claudia Canzonetta, Elisavet Stathaki, Stefania Gimelli, Frederique Sloan‐Bena, Robert Abrehart, Pollyanna Goh, Shuhui Lim, Chiara Baldo, Franca Dagna‐Bricarelli, Saad Hannan, Martin Mortensen, David Ballard, Denise Syndercombe Court, Noemi Fusaki, Mamoru Hasegawa, Trevor G. Smart, Cleo Bishop, Stylianos E. Antonarakis, Jürgen Groet, Dean Nizetic

**Affiliations:** ^1^ The Blizard InstituteBarts and The London School of MedicineLondonUnited Kingdom; ^2^ Stem Cell Laboratory, National Centre for Bowel Research and Surgical Innovation, Queen Mary University of LondonLondonUnited Kingdom; ^3^ The LonDownS Consortium, Wellcome TrustLondonUnited Kingdom; ^4^ Department of Genetic Medicine and DevelopmentUniversity of Geneva Medical SchoolGenevaSwitzerland; ^5^ Service of Genetic Medicine, University Geneva HospitalsGenevaSwitzerland; ^6^ Lee Kong Chian School of Medicine, Nanyang Technological UniversitySingaporeSingapore; ^7^ Human Genetics Laboratory, Galliera HospitalGenoaItaly; ^8^ Liguria Department of GeneticsGenoaItaly; ^9^ Department of Neuroscience, Physiology and PharmacologyUniversity College LondonLondonUnited Kingdom; ^10^ Department of Forensic and Analytical ScienceKing's CollegeLondonUnited Kingdom; ^11^ Precursory Research for Embryonic Science and Technology, Japan Science and Technology AgencySaitamaJapan; ^12^ DNAVEC CorporationIbarakiTokyoJapan

**Keywords:** Down syndrome, Neurodegeneration, Neurogenesis, Neuronal differentiation

## Abstract

Trisomy 21 (T21), Down Syndrome (DS) is the most common genetic cause of dementia and intellectual disability. Modeling DS is beginning to yield pharmaceutical therapeutic interventions for amelioration of intellectual disability, which are currently being tested in clinical trials. DS is also a unique genetic system for investigation of pathological and protective mechanisms for accelerated ageing, neurodegeneration, dementia, cancer, and other important common diseases. New drugs could be identified and disease mechanisms better understood by establishment of well‐controlled cell model systems. We have developed a first nonintegration‐reprogrammed isogenic human induced pluripotent stem cell (iPSC) model of DS by reprogramming the skin fibroblasts from an adult individual with constitutional mosaicism for DS and separately cloning multiple isogenic T21 and euploid (D21) iPSC lines. Our model shows a very low number of reprogramming rearrangements as assessed by a high‐resolution whole genome CGH‐array hybridization, and it reproduces several cellular pathologies seen in primary human DS cells, as assessed by automated high‐content microscopic analysis. Early differentiation shows an imbalance of the lineage‐specific stem/progenitor cell compartments: T21 causes slower proliferation of neural and faster expansion of hematopoietic lineage. T21 iPSC‐derived neurons show increased production of amyloid peptide‐containing material, a decrease in mitochondrial membrane potential, and an increased number and abnormal appearance of mitochondria. Finally, T21‐derived neurons show significantly higher number of DNA double‐strand breaks than isogenic D21 controls. Our fully isogenic system therefore opens possibilities for modeling mechanisms of developmental, accelerated ageing, and neurodegenerative pathologies caused by T21. Stem Cells *2015;33:2077–2084*

## Introduction

Trisomy 21 (T21), Down Syndrome (DS) is the most common genetic cause of intellectual disability and dementia with rising global prevalence 1. Several phenotypes have been observed at molecular and cellular levels that reproduce in primary tissues from human individuals with T21. Some of these cellular phenotypes directly map onto clinical components of DS; these include intellectual disability, defects in cognitive development and age‐related cognitive decline, Alzheimer's disease‐like dementia, epilepsy, congenital heart defect, childhood leukemia, and others 2, 3, 4, 5, 6, 7, 8, 9, 10, 11, 12. Modeling DS is beginning to yield pharmaceutical therapeutic interventions for amelioration of intellectual disability which are currently being tested in clinical trials. New drugs could be identified by high throughput screening of chemical libraries using cellular assays, and therefore well‐controlled cellular model systems are required. In order to eliminate effects of wide phenotypic differences among individuals with DS, the requirement for many experimental purposes has become the use of an isogenic induced pluripotent stem cell (iPSC) model for DS, where the sole difference between iPSC lines is the presence of the third chromosome 21.

Several recent iPSC models of DS have been developed. All of these (with one exception) used integrational reprogramming. All of these reprogrammed cells were derived from fetal, neonatal, or 1 year old infant DS; actually, three studies 13, 14, 15 used the same iPSC lines as a starting point 16. Some of these studies report defects in neural progenitor cell (NPC) proliferation 17, neurogenesis 13, gliogenesis, and neurite outgrowth 18, others defects in synaptic morphology and function, mitochondrial dysfunction 19, and increased amyloid deposition 15. Other models show increased propensity to generate hematopoietic precursors and increased multilineage myeloid hematopoiesis potential 14, 20. Most of these results have been generated on nonisogenic comparisons, with isogenic lines either serendipitously generated in cell culture 14, 19 or using an ingenious but with complex and laborious approach to silence the third chromosome 21 13, 21. In some cases, nonisogenic lines were pooled with one isogenic line 14, 19. In one study, a unique case of heterokaryotypic twin fetal cells was exploited 17. None of these papers reported a genome‐wide high‐resolution array‐comparative genomic hybridization (aCGH) analysis of the resulting iPSCs for the rigorous measurement of the artificial copy‐number rearrangements known to frequently occur during the reprogramming process.

Here we present the first iPSC DS model which is both nonintegrationally reprogrammed and fully isogenic and the first derived from an adult individual with DS which is a constitutional mosaic. Furthermore, we verify the high level of genome integrity of the resulting iPSCs by showing a very low number of reprogramming rearrangements as assessed by a high‐resolution whole genome aCGH, and we make entirely isogenic comparisons of three trisomic and three disomic iPSC lines derived from this model. This approach minimizes the influence of any copy number fluctuations additional to T21 and eliminates genotypic difference noise, allowing the “clean” detection of T21‐causing effects. Our model reproduces several T21 cellular pathologies seen in primary human DS cells but hitherto not reported in iPSC models, such as abnormalities in mitochondrial number and size and an increase in DNA double‐strand breaks in neurons.

## Materials and Methods

Detailed methods are shown in Supporting Information online.

## Results

**Figure 1:**
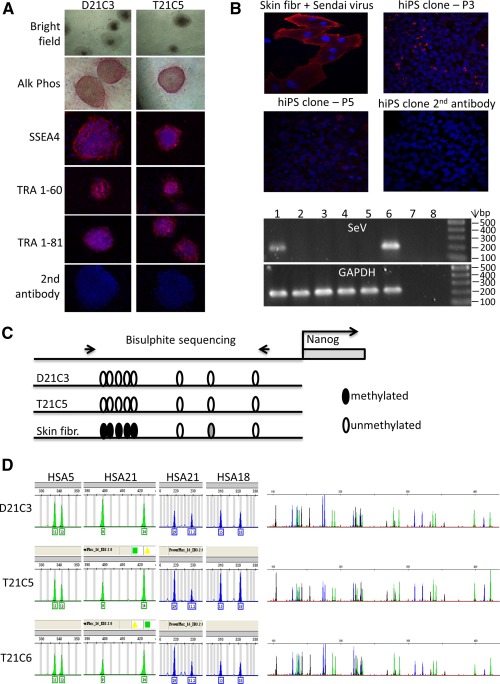
Isogenic iPSC model of Down Syndrome generated by reprogramming primary human skin fibroblasts from an adult individual with mosaic Down Syndrome, using a temperature‐sensitive Sendai virus. **(A):** Images of undifferentiated iPSC colonies from two clones (D21C3, T21C5) after three passages; bright‐field microphotographs and alkaline phosphatase expression. Further images after immunohistochemistry for pluripotency markers (SSEA4, TRA 1–60, and TRA 1–81). **(B):** Spontaneous elimination of the temperature sensitive, nonintegrating Sendai virus (Ts‐SeV) from iPSC cells through routine passaging. Primary human skin fibroblasts were infected with the Ts‐SeV for positive control and were stained with an antibody against the SeV protein HN‐IL4.1 alongside the iPSC colonies at the indicated passage numbers (P[n]). The agarose gel shows amplification products after reverse transcriptase polymerase chain reaction (PCR), using SeV (and GAPDH) specific primers (1) D21C3 P5, (2) T21C5 P7, (3) T21C5 P10, (4) D21C3 P10, (5) untransfected fibroblasts, (6) SeV infected fibroblasts P0, (7) SeV infected fibroblasts P0—reverse transcriptase, (8) H_2_O C: Demethylation of endogenous *NANOG* promoter following reprogramming: bisulfite sequencing analysis of eight CpG dinucleotides in the promoter region of *NANOG* using genomic DNA isolated from iPSC D21C3 and T21C5, compared to genomic DNA isolated from the primary mosaic DS skin fibroblasts that were used for reprogramming. **(D):** (Left hand panels): Graphs showing semiquantitative microsatellite PCR analysis for two chromosome 21 (HSA21) markers and for markers from two euploid chromosomes (HSA5, 18) using genomic DNA isolated from iPSC clones. Clones T21C5 and T21C6 are trisomic and Clone D21C3 disomic for HSA21. (Right hand panels): Whole genome microsatellite fingerprint of genomic DNA isolated from iPSC clones, demonstrating that they are isogenic. Abbreviations: Alk Phos, alkaline phosphatase; hiPS clone, human induced pluripotent stem clone; SeV, Sendai Virus.

We generated the iPSCs by nonintegration reprogramming using temperature‐sensitive Sendai virus 22 from primary skin fibroblasts from a young adult diagnosed with constitutional mosaicism for DS (strategy illustrated in Supporting Information Fig. S1). We isolated individual clones (labeled as C[number]), expanded them, and confirmed that they tested positive for alkaline‐phosphatase expression, and the presence of markers of pluripotency (Fig. 1A). The Sendai virus was efficiently removed after 7–10 passages (Fig. 1B). Demethylation of the endogenous *NANOG* promoter in the iPSCs, compared to the parental skin fibroblasts, was established via bisulfite sequencing (Fig. 1C). Individual clones were analyzed by rigorous whole‐genome microsatellite DNA fingerprinting, which established the presence of clones with T21, and euploid genome (D21), which are otherwise isogenic (Fig. 1D). In a preliminary RNA‐seq experiment, the isogenic iPSCs show an expected increase in transcript levels for the majority of HSA21 genes (not shown). The genome integrity of the resulting iPSCs is of a high level, as was assessed by high‐resolution, whole genome aCGH (Supporting Information Fig. S2). The supernumerary HSA21 is intact and complete in both analyzed trisomic lines (T21C5 and T21C6), and T21 is stable for at least 17–19 passages, which is as far as we tested for the presence of T21 (Supporting Information Fig. S2A). After filtering out the copy number variations (CNVs) that occur commonly in healthy individuals (using comparison to the Database of Genomic Variation) we made an in silico comparison with the published survey of genome‐rearrangement artefacts in iPSC generated by conventional integration‐reprogramming 23. Selecting only events at the same aCGH resolution and the same passage number as in our study, we detected a significantly lower number of uncommon CNVs, affecting a significantly lower number of genes in our lines, relative to those generated by classic integrational reprogramming methods (Supporting Information Fig. S2B, S2C). In compliance with international guidelines for iPSC nomenclature 24 we name these iPSCs: NIZEDSM1iD21‐C3, ‐C7, and ‐C9 for the disomic lines, respectively, and NIZEDSM1iT21‐C5, ‐C6, ‐C13 for the trisomic lines, respectively (henceforward abbreviated to D21C3, D21C7, D21C9, T21C5, T21C6, and T21C13). Microsatellite DNA fingerprinting was repeated at later passages and confirmed that trisomy 21 is retained through routine passaging (Supporting Information Fig. S2D). The isogenic DS iPSC clones can differentiate into cell lineages of all three embryonic layers both in vitro and in vivo (Supporting Information Fig. S3).

After 45 days in culture, using neuronal differentiation via neuro embryoid body (NEB) protocol (Supporting Information Methods), both disomic and trisomic neuronal differentiation cultures were able to produce mature looking neurons expressing βIII‐tubulin, the inhibitory neurotransmitter GABA, as well as presynaptic and postsynaptic markers of excitatory synapses, PSD95 and VGlut (Supporting Information Fig. S4A, S4B, respectively). In order to accelerate neuronal differentiation and improve yields, we applied a directed neuronal differentiation protocol using dual SMAD inhibition (Noggin and SB431542), combined with stimulation of retinoid signaling and the addition of the Sonic Hedgehog agonist purmorphamine 25. Both trisomic and disomic lines were able to produce electrophysiologically active neurons (Supporting Information Fig. S4C) that supported spontaneous action potential firing and functional whole‐cell current responses to saturating concentrations of externally applied GABA and glycine, observed in neurons from both D21C3 (at 28 days) and T21C5 lines (at 40 days). In order to gain an approximate estimate of the proportion of neurons likely to fire spontaneous action potentials, we used Ca^2+^ imaging and observed multiple Ca^2+^ transients, which would be indicative of regenerative spontaneous activity in accord with the firing of spontaneous action potentials (Supporting Information Fig. S5A, S5B). Quantification of the numbers of neurons exhibiting calcium transients over the 1 minute time course showed no significant difference between D21 and T21 neurons (Supporting Information Fig. S5B). In addition, under voltage‐clamp conditions, we identified neuronal sensitivities to GABA and glycine which indicates the cell surface expression of functional GABA_A_ and glycine receptors. This was apparent in most cells tested, with no difference noted between T21 and D21 cells (not shown).

To examine early stages of iPSC differentiation an EB protocol was adopted for both hematopoietic and neuronal differentiation, modifying the published method 26. For hematopoietic EBs (HEBs) 3,000 live cells in single cell suspension were allowed to aggregate in a 96‐well. Imaging and analysis of HEBs after 5 days showed that T21 HEBs were significantly larger than the euploid controls (Supporting Information Fig. S6A). This increase in HEB size was caused by an increase in the cell numbers (Supporting Information Fig. S6B), which at this stage is predicted to be early hematopoietic mesoderm precursors. A similar result was obtained with early T21 mesodermal colonies derived from transchromosomic mouse embryonic stem cells 7. Although the T21 HEBs gave a higher proportion of CD34+ cells (hematopoietic stem/progenitor lineages) on both days 12 and 18 of further differentiation (not shown), there was no statistically significant difference. By contrast, NPCs derived from NEBs showed a reduction in proliferation rate. The cumulative population doublings of trisomic cells was significantly reduced during the expansion of NPCs (Supporting Information Fig. S6C). Increased cell death is unlikely to be the cause of the observed decrease population cell doubling, because the proportion of nonviable cells in the same counts shows no increase in T21 NPCs (Supporting Information Fig. S6D).

**Figure 2:**
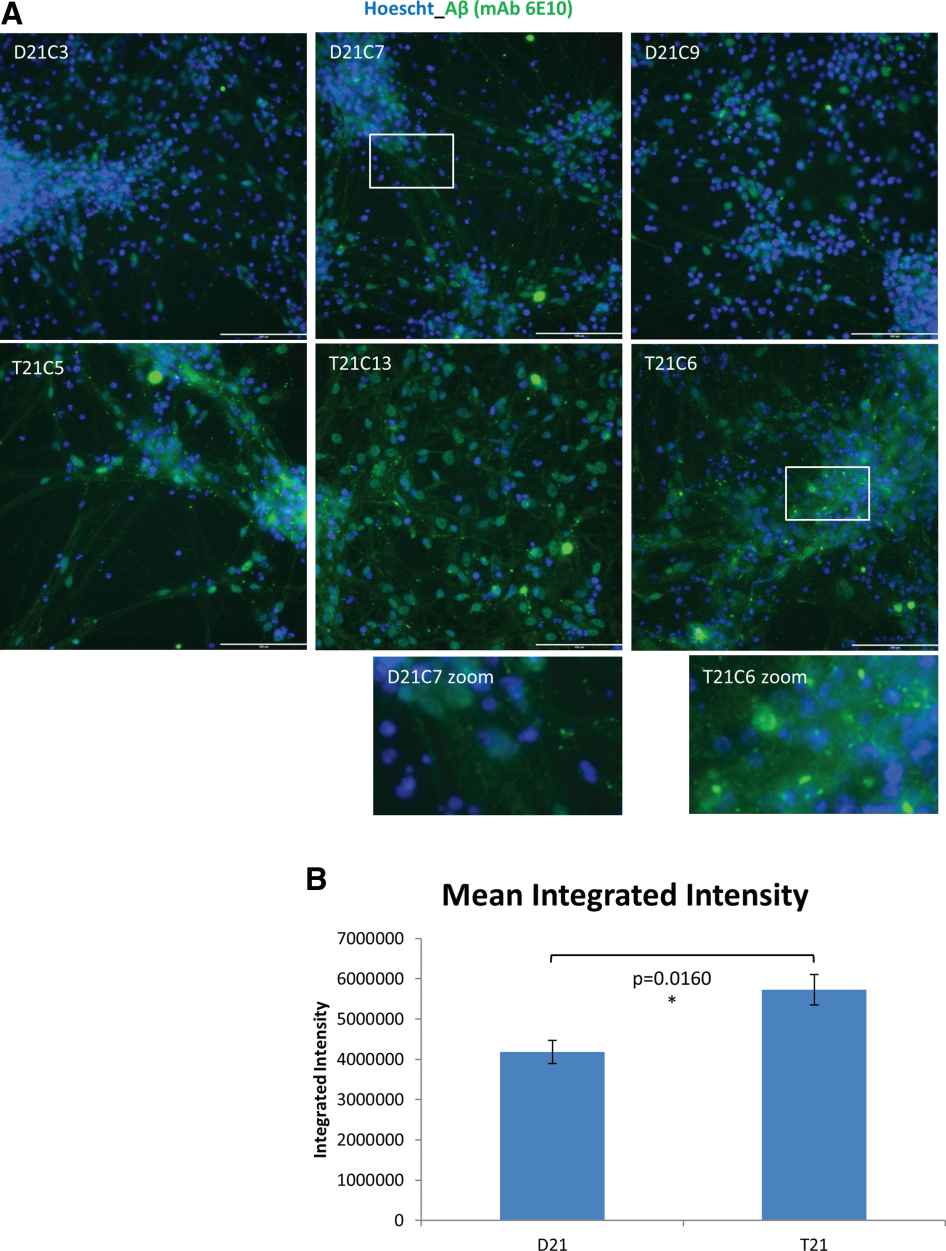
Trisomy 21 causes an increase in β‐amyloid containing material in and around neurons generated from iPSCs. **(A):** Neurons were generated from iPSCs over a 60‐day differentiation protocol. Cells were then fixed and stained with an anti‐amyloid peptide antibody (6E10), which is reactive to amino acids 1–16 in β‐amyloid but detects all APP polypeptide forms that contain the epitope. Nuclei were labeled with Hoechst. Scale bar = 100 μm. **(B):** Quantification of the integrated intensity for the 6E10 stain shows an increase of APP expression in T21 neurons compared to the isogenic D21 neurons. Image capture and quantification were performed using automated multiparametric analysis on the ImageXpress Micro XL (Molecular Devices) wide‐field high content imaging system, and data were analyzed using MetaMorph software. Three wells per cell line and a minimum of 6,000 cells per well were analyzed. Student's *t* test, error bars SEM. Visually, T21 neurons appear to also show more 6E10‐reactive aggregates (not quantitated). Zoomed‐in images for T21C6 and D21C7 are shown at the same size and magnification.

iPSC‐derived neurons were analyzed after 60 days of differentiation following the dual SMAD inhibition protocol (mentioned previously, but here minus purmorphamine) that generates multiple classes of projection neurons from different cortical layers 25. We observed the typical morphology of neurogenic cortical rosettes as published 27 and confirmed that after 60 days neuronal differentiation, >95% of cells were Tuj1+ (not shown). As this protocol generated 100% excitatory glutamatergic neurons 25, 27, and as no differences in the composition of cortical layer subpopulations were observed between DS and normal human iPSCs 15, we think it is unlikely that difference in cell type profiles would be responsible for the differences between T21 and D21 observed in our results, although we cannot fully rule out this possibility. Immunostaining of fixed neurons with an antibody (6E10) raised against the epitopes in the Aβ‐peptide, but reactive to all proteolytic fragments of the amyloid precursor protein that contain this epitope, revealed an increase in total amyloid staining in trisomic neurons compared to isogenic euploid controls (Fig. 2A, 2B). This demonstrates that increased amyloid production can be successfully modeled in these cells. Trisomic neurons also appear to show an increase in size and number of 6E10‐reactive discreet punctiform aggregates (not quantified, Fig. 2B zoom). Our staining does not permit us to conclude if these aggregates are intracellular or extracellular.

**Figure 3:**
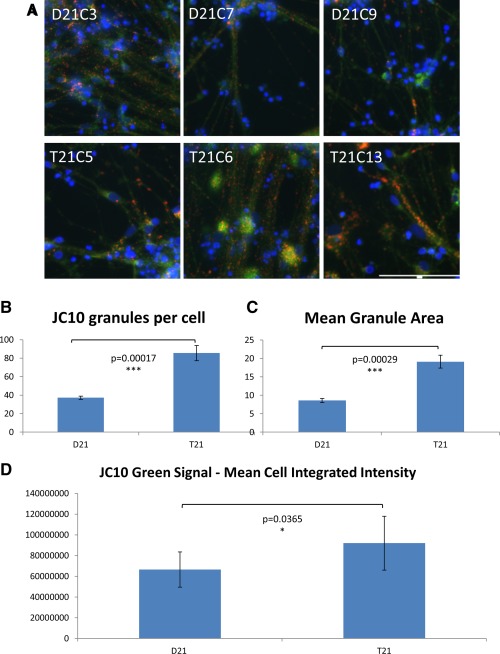
Trisomy 21 results in an increase in size and number of mitochondria in neurons generated from induced pluripotent stem cells (iPSCs). **(A):** Neurons were generated from iPSCs over a 60‐day differentiation protocol. Live cells were then loaded with JC‐10 to assess mitochondrial membrane potential. Healthy mitochondria are labeled in red, while green cytoplasmic staining indicates that JC‐10 is diffusing out of the mitochondria due to decreased mitochondrial membrane potential. Representative images for each cell line are shown. Image capture and quantification were performed using automated multiparametric analysis on the ImageXpress Micro XL (Molecular Devices) wide‐field high content imaging system, and data were analyzed using MetaMorph software. A total of four wells and a minimum of 1,500 cells per cell line were imaged and analyzed. Scale bar = 100 μm (identical scale for all images). **(B):** Quantification of the number of mitochondria per cell, and **(C)** the mean mitochondrial area show that both are increased in T21 neurons compared to the isogenic D21 neurons. **(D):** Quantification of the integrated intensity for the green signal generated by JC‐10 shows decreased mitochondrial membrane potential in T21 neurons compared to the isogenic D21 neurons. Student's *t* test, error bars SEM.

To assess mitochondrial membrane potential in neurons, live cells were labeled with JC‐10 (Fig. 3A). In healthy cells JC‐10 selectively accumulates in the mitochondria and forms aggregates with a characteristic fluorescent emission at 590 nm (orange/red). If mitochondrial membrane potential is decreased (due to damaged/unhealthy cells) JC‐10 monomers are formed, which are released into the cytoplasm, resulting in a shift to green emission at 525 nm. An increase in both the size and number of mitochondria were observed in the trisomic neurons (Fig. 3B, 3C), consistent with a previous study in primary T21 neurons which showed “generalized perturbations” in T21 mitochondrial structure and function, including a more fragmented mitochondrial network 9. Trisomic neurons also showed a decreased mitochondrial membrane potential, as evidenced by the increase in green cytoplasmic staining with JC‐10 (Fig. 3D).

**Figure 4:**
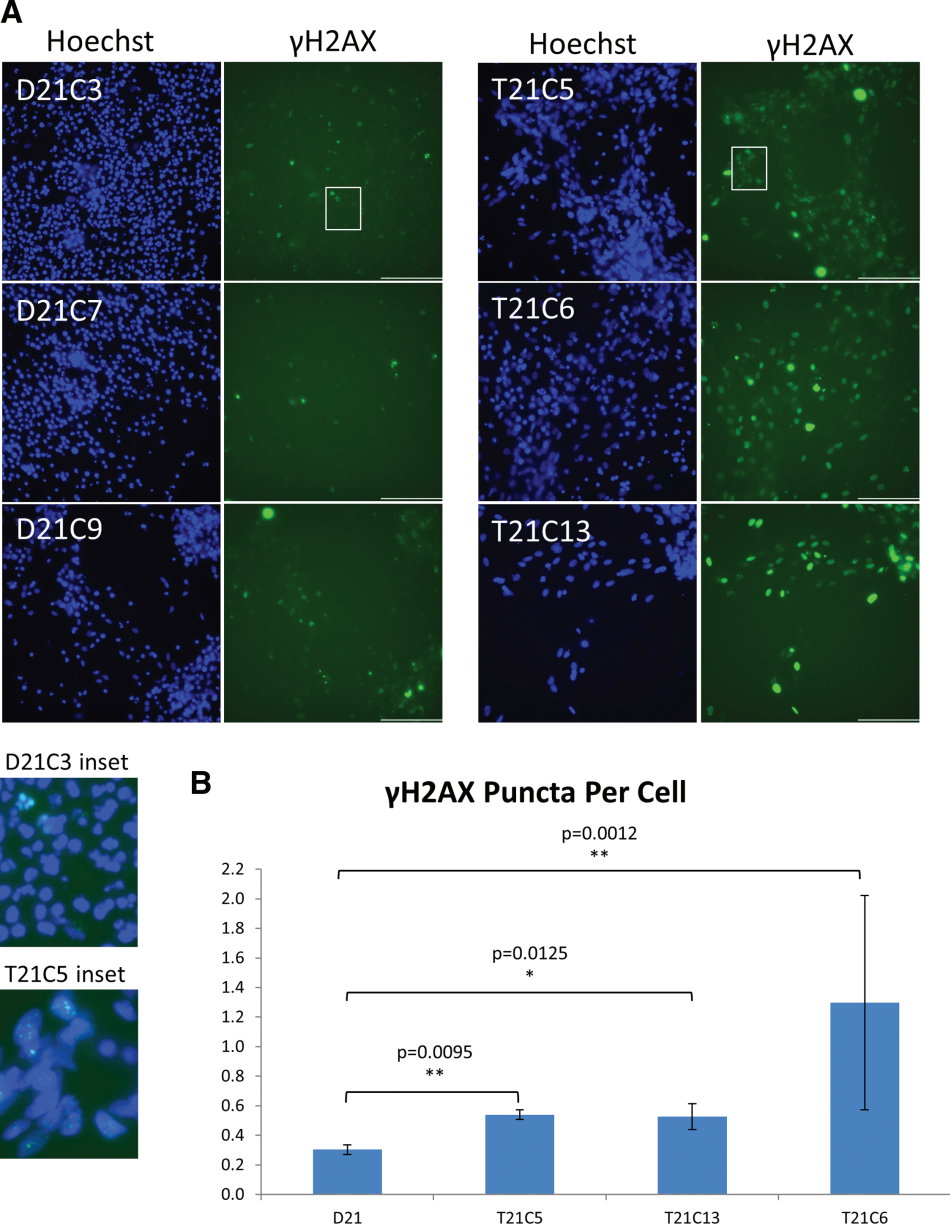
Trisomy 21 causes an increase in DNA damage in neurons generated from induced pluripotent stem cells (iPSCs). **(A):** Neurons were generated from iPSCs over a 60‐day differentiation protocol. Cells were then fixed and stained with a γH2AX antibody to detect DNA double‐strand breaks. Nuclei were labeled with Hoechst. Scale bar = 100 μm. Enlarged insets for D21C3 and T21C5 double stained with Hoechst and γH2AX antibody are shown as examples below main images. **(B):** The number of γH2AX puncta per cell is significantly increased in T21 neurons compared to the isogenic D21 neurons. Image capture and quantification were performed using automated multiparametric analysis on the ImageXpress Micro XL (Molecular Devices) wide‐field high content imaging system, and data were analyzed using MetaMorph software. Three wells per cell line and a minimum of 6,000 cells per well were analyzed. Student's *t* test, error bars SEM.

Having shown that T21 neurons have a deficit in mitochondrial function similar to primary human T21 neurons in vitro, we anticipate that reactive oxygen species (ROS) production is increased in T21 neurons. Increased ROS leads to increased DNA damage, as measured by the proportion of γH2AX foci, an in vitro marker of ageing, shown increased in primary fibroblasts from old, compared to young humans 28, and in primary neurons from old, compared to young mice 29. We assessed the number of γH2AX foci (Fig. 4A). Fully automated quantification of approximately 18,000 cells per cell line showed a significantly increased number of γH2AX puncta per cell in T21 neurons (Fig. 4B).

## Discussion

In conclusion, we report a first nonintegration‐reprogrammed isogenic and high genomic fidelity iPSC model from an adult with mosaic DS. The model reproduces several differentiation, ageing, and neurodegeneration‐related cellular phenotypes associated with DS pathology and attributable solely to T21 as a cause.

Particularly intriguing are two observations (Figs. 3, 4) that open a whole set of new interesting mechanistic questions. Increased number of mitochondria in neurons could be related to increased mitochondrial fragmentation observed in primary DS cortical neurons in culture 9. On the other hand, it is possible that T21 mitochondria in DS neurons, which are high consumers of energy exclusively derived from oxidative metabolism, are hypofunctional (as has been observed in primary DS cortical neurons in culture 9), and the demand for more energy increases mitochondrial biogenesis. These hypotheses remain to be tested. Increased number of DNA double‐strand breaks in T21 neurons (Fig. 4) could be further exploited as a cellular marker of accelerated ageing observed in DS 10, 30. Alternatively, more genomic instability in the nuclei of postmitotic T21 neurons (such as transposition events) 31 could also explain this observation.

Currently, several interdisciplinary consortia have been organized to study DS genetics and cellular models integrated with the assessment of the adult population with DS for neurocognitive function, age‐related decline, presence or absence of dementia and other comorbidities (e.g., see http://www.ucl.ac.uk/london‐down‐syndrome‐consortium, http://www.psychiatry.cam.ac.uk/ciddrg/research/dementia‐in‐downs‐syndrome‐dids/). It is therefore important to verify that cellular phenotypes under‐pinning DS pathology can also be reproduced in iPSCs derived from an adult individual with DS, as this had not been reported so far.

Up to 75% of constitutionally T21 concepti spontaneously die in utero 10, 32 suggesting that in random allelic variation, the presence of a third copy of HSA21 has a 75% probability to cause severe phenotypes that are normally missed. In constitutional mosaicism, T21 with an otherwise deleterious genotype could be rescued by a significant (>50%) presence of normal (D21) cells, which results in varying intensity of clinical DS defects, often not correlating with the percentage of trisomic cells in tissues. This presents with a theoretical (but so far never explored) rationale for T21/D21 cells derived from a constitutionally mosaic DS individual to show more contrasting differences in cellular phenotypes, than comparisons between cells of liveborn 100% trisomic individuals. Such phenotypes are more likely to be robust, reproducible, and therefore more amenable to developing into high throughput screening assays.

## Author Contributions

A.M. and J.G.: conception and design, collection and assembly of data, data analysis and interpretation, manuscript writing, and final approval of manuscript; A.L.: conception and design, collection and assembly of data, data analysis and interpretation, and manuscript writing; C.C.: conception and design, collection and assembly of data, and data analysis and interpretation; E.S., S.G., and R.A.: collection of data; F.S.‐B., P.G., S.H., M.M., D.B., and D.S.‐C.: collection of data and data analysis and interpretation; S.L.: data analysis and interpretation; C.Ba. and F.D.‐B: provision of study material or patients; N.F. and M.H.: provision of study material; T.S: assembly of data and data analysis and interpretation; C.Bi.: collection and assembly of data, data analysis and interpretation, manuscript writing, and final approval of manuscript; S.A.: financial support, data analysis and interpretation, manuscript writing, and final approval of manuscript; D.N.: conception and design, financial support, collection and assembly of data, data analysis and interpretation, manuscript writing, and final approval of manuscript.

## Disclosure of Potential Conflicts of Interest

The authors indicate no potential conflicts of interest

## Supporting information

Supplementary Information Figure S1

Supplementary Information Figure S2

Supplementary Information Figure S3

Supplementary Information Figure S4

Supplementary Information Figure S5

Supplementary Information Figure S6

Supplementary Information Methods
