# Supplementary figures and images for: Brief Report: Isogenic Induced Pluripotent Stem Cell Lines From an Adult With Mosaic Down Syndrome Model Accelerated Neuronal Ageing and Neurodegeneration
Source: Stem Cells. 2015 May 21;33(6):2077–84. doi: 10.1002/stem.1968 (PMC4737213; doi:10.1002/stem.1968)

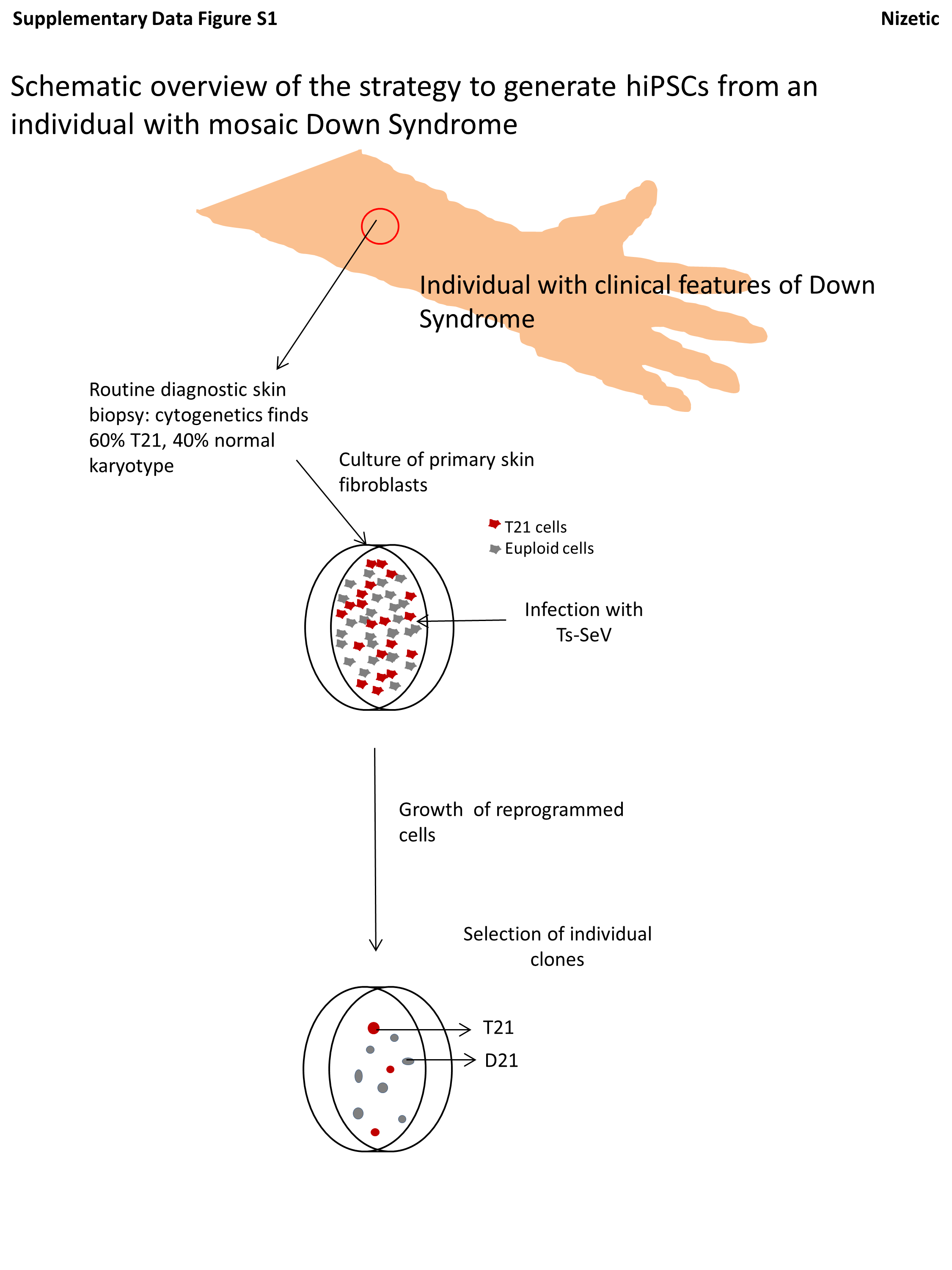

Supplement: Supplementary file 1 — Supplementary Information Figure S1 [file STEM-33-2077-s001.tif]

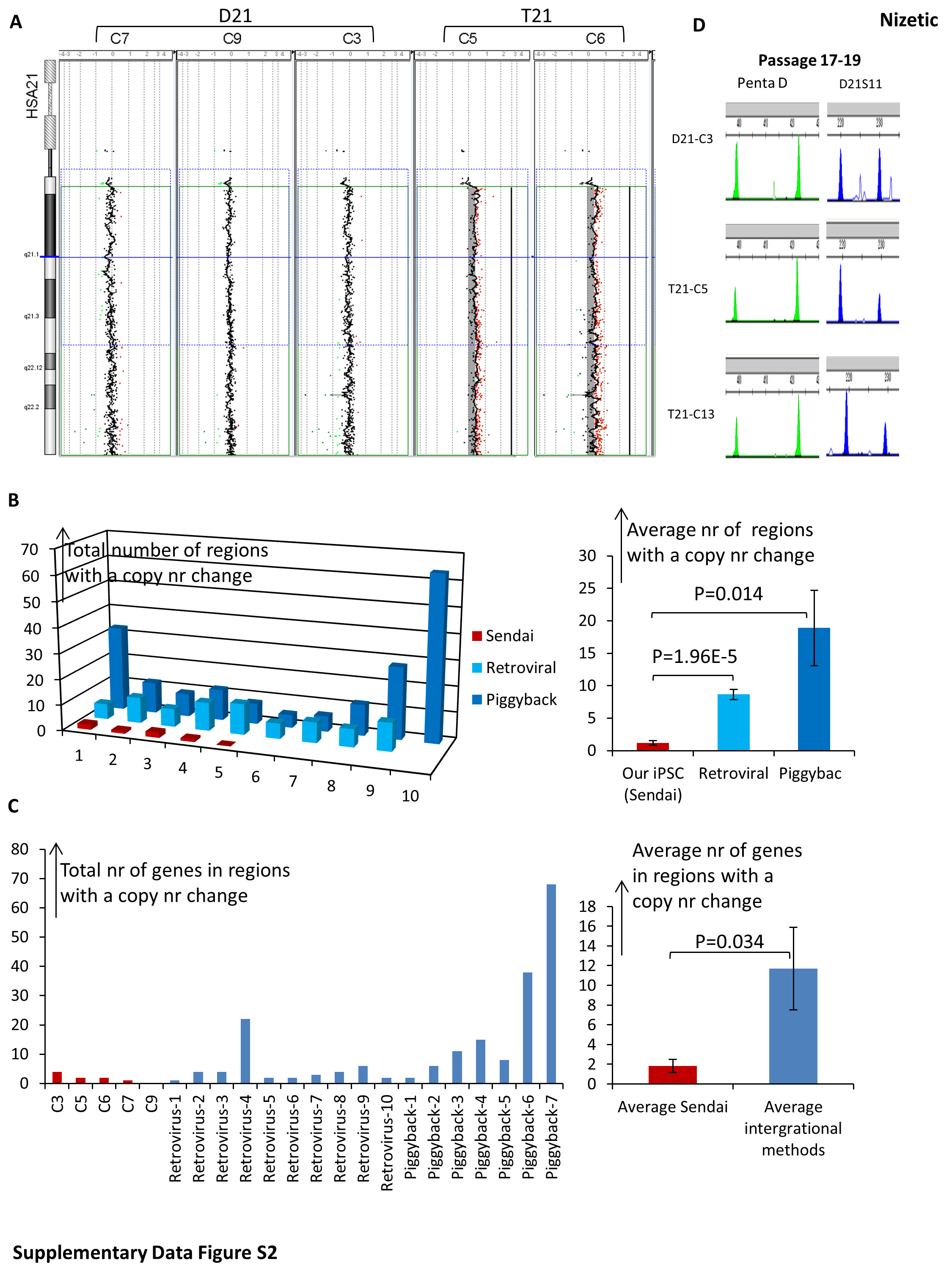

Supplement: Supplementary file 2 — Supplementary Information Figure S2 [file STEM-33-2077-s002.tif]

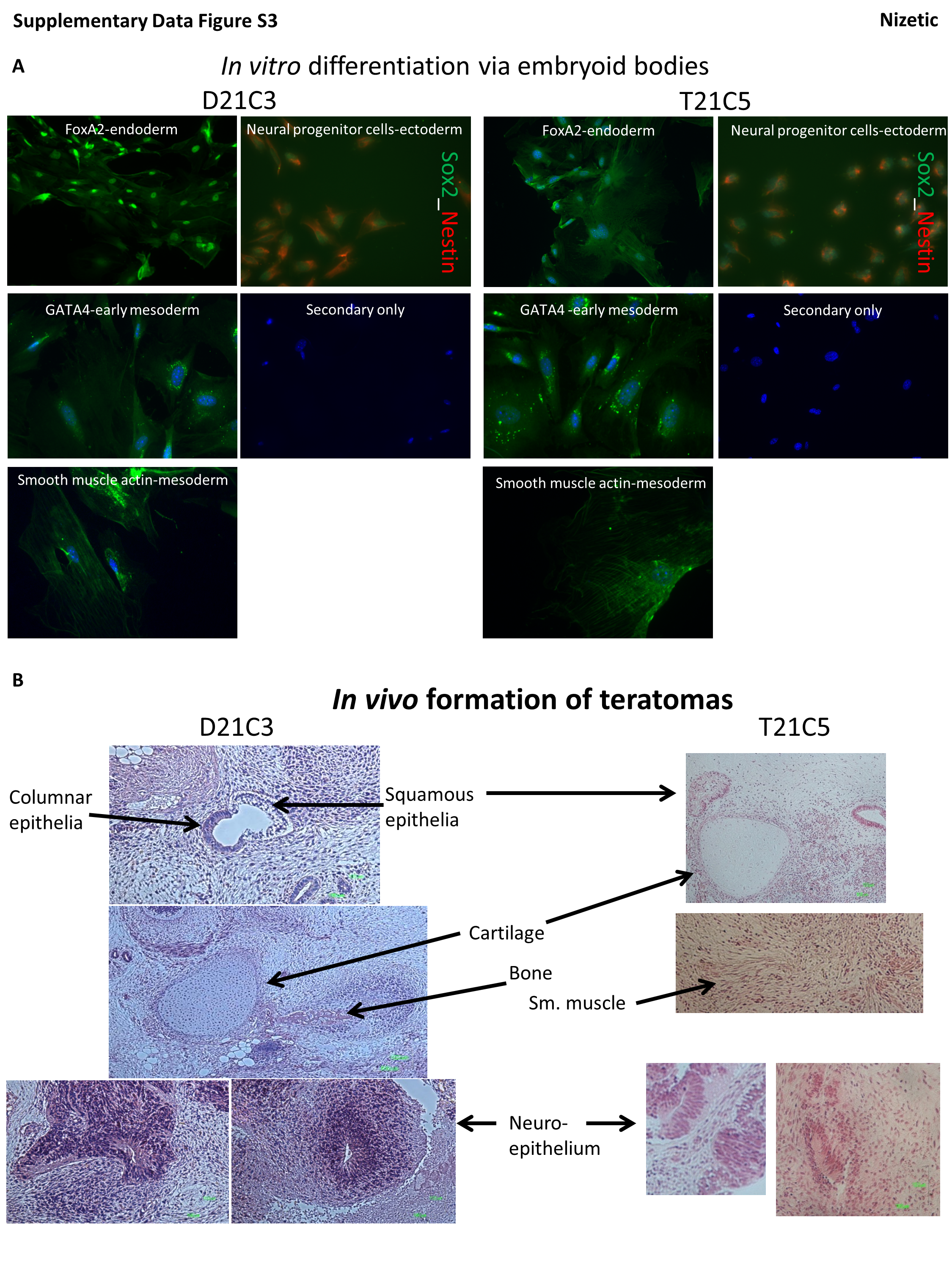

Supplement: Supplementary file 3 — Supplementary Information Figure S3 [file STEM-33-2077-s003.tif]

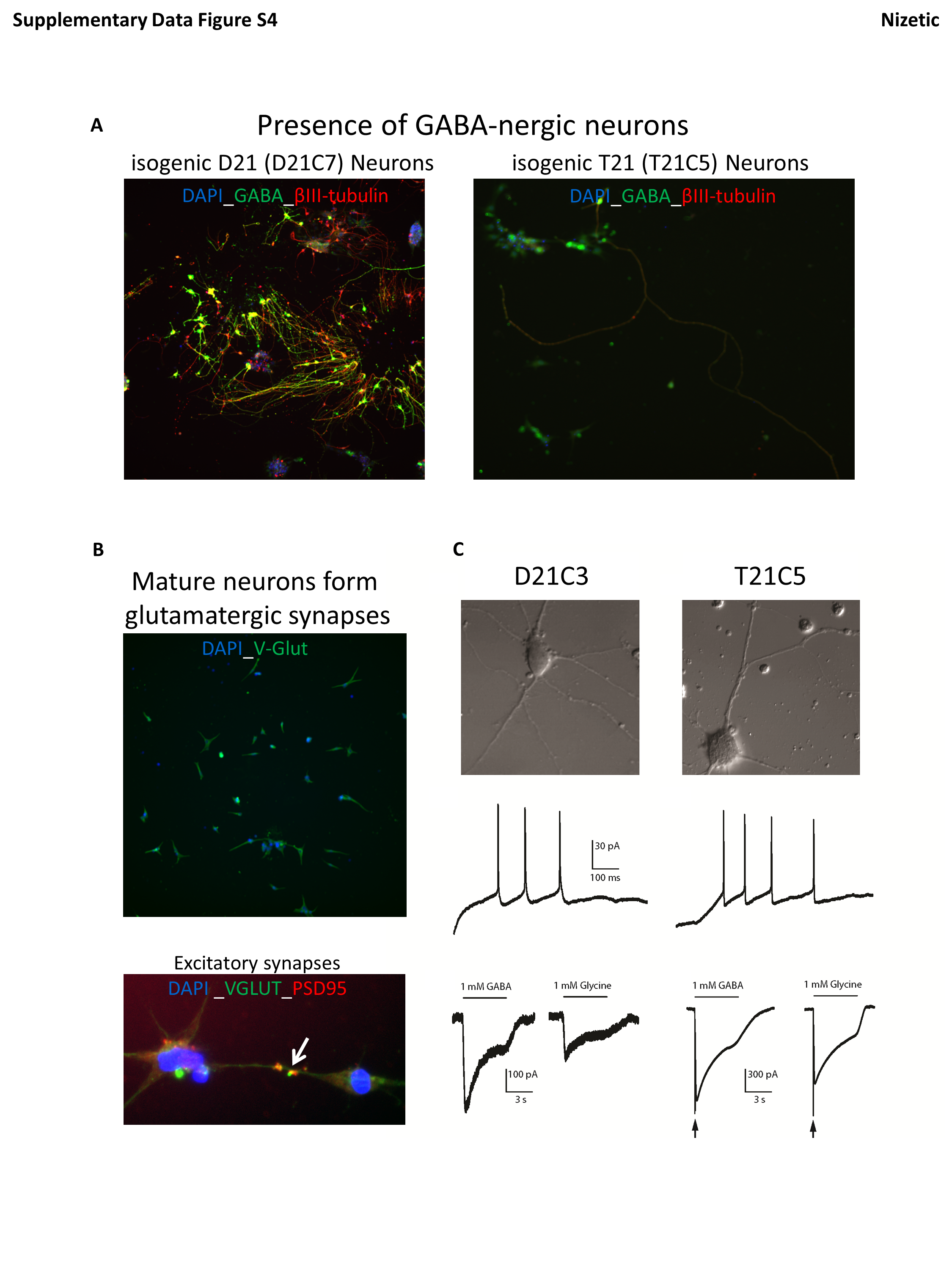

Supplement: Supplementary file 4 — Supplementary Information Figure S4 [file STEM-33-2077-s004.tif]

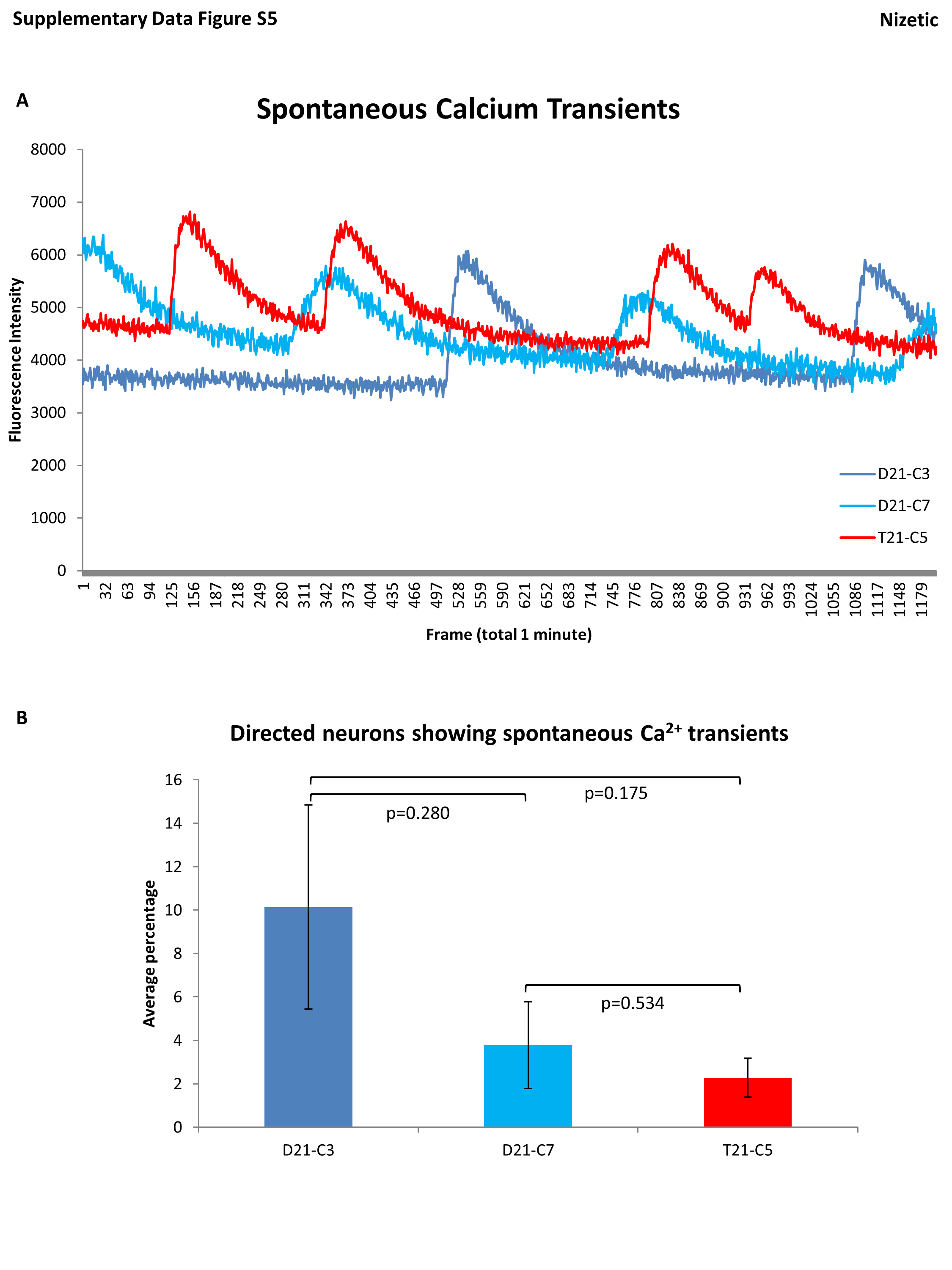

Supplement: Supplementary file 5 — Supplementary Information Figure S5 [file STEM-33-2077-s005.tif]

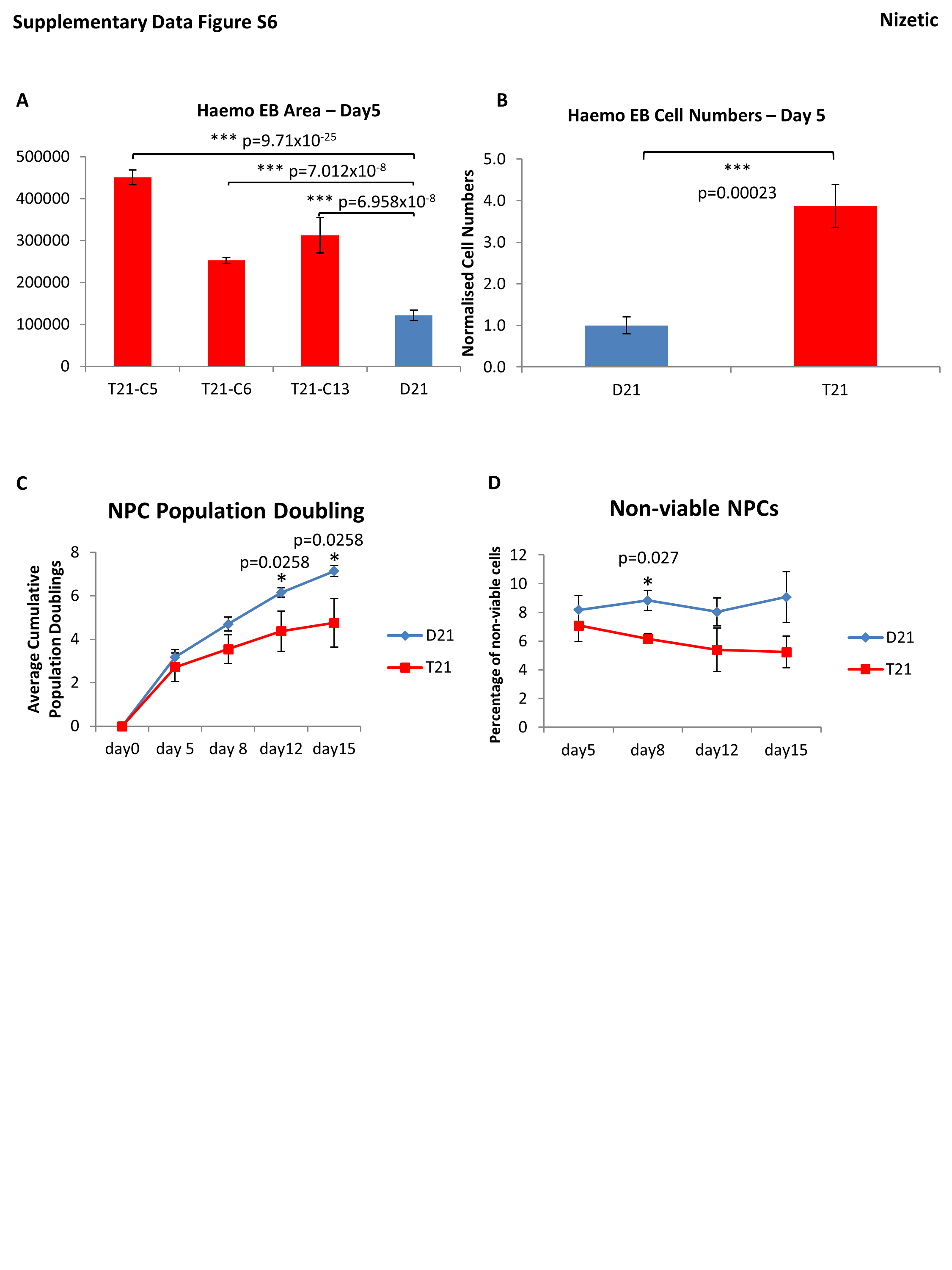

Supplement: Supplementary file 6 — Supplementary Information Figure S6 [file STEM-33-2077-s006.tif]
