# Supplementary material for: Brief Report: Isogenic Induced Pluripotent Stem Cell Lines From an Adult With Mosaic Down Syndrome Model Accelerated Neuronal Ageing and Neurodegeneration
Source: Stem Cells. 2015 May 21;33(6):2077–84. doi: 10.1002/stem.1968 (PMC4737213; doi:10.1002/stem.1968)
Supplement: Supplementary file 7 — Supplementary Information Methods [file STEM-33-2077-s007.docx]

**Supplementary Methods**

**iPSC generation characterization and maintenance**

Consented donated skin tissue from a young adult with mosaic Down Syndrome was collected by the Galliera Genetic Bank, where mosaicism (60% trisomy 21, 40% normal karyotype) was detected by routine diagnostic cytogenetics. Primary skin fibroblasts were used and stored in The Blizard Institute under Human Tissue Authority Licence 12199. These cells were cultured in RPMI with 15% FCS at 37°C, 5% CO_2_. Mosaic primary fibroblasts were reprogrammed in the 8th passage using temperature-sensitive Sendai Virus (Ts-SeV) reagent delivering the OCT4, KLF4, SOX2 and MYC transcripts, as described^1^. A total of 12 Alkaline Phosphatase positive lines were established, and of these, 2 had trisomy 21, and 10 had disomy 21, as determined by quantitative microsatellite analysis. A large number of markers from other chromosomes confirmed that all have the same genotype (isogenic). Line T21C13 was a very-early-passage, single-colony sub-clone of T21C5, with independent culture history for most of its use. iPSC lines were maintained in HESC medium (DMEM/F12, knock out serum, 2 mM Glutamine, 1x Penicillin/Streptomycin, 1x Non-essential amino acids (NEAA), 100 µM Beta mercaptoethanol, FGF-2 (50 ng/ml)), on mouse embryonic fibroblasts (grown on 0.1% w/v gelatin). iPSC clones were passaged using dispase (1 mg/ml, for 15 min, 37°C), lifted by gentle pipetting with HESC medium containing 10µM ROCK inhibitor Y-27632 (Tocris Bioscience).

**Bisulfite sequencing**

1μg of genomic DNA was extracted from human iPSCs and fibroblasts and digested with *HindIII* (NEB, Hitchin, UK). Buffer exchange was established with microcon columns (YM-100, Millipore, Watford, UK). DNA was resuspended in 14.4 μl H_2_O, and 1.6 μl of NaOH was added. This was incubated for 20 minutes at 42°C. 8μl was transferred into a new tube and bisulfite was added, 86.5μl of 2.3M Na_2_S_2_O_5_ and 5μl of 10 mM hydroquinone, and incubated at 50°C for 16 hours. Buffer exchange was established with microcon columns, after which 350μl 0.1M NaOH was added, centrifuged and flow through removed. Two additional washes with 350μl H_2_O were performed, after which the DNA was resuspended in 10μl H_2_O. 1μl of bisulfite modified DNA was amplified (95°C for 7.5 minutes; (95°C for 30 seconds; 60°C for 30 seconds; 72°C for 30 seconds) 35x, 72°C for 9.5 minutes. Primers: Nanog-F: TGGTTAGGTTGGTTTTAAATTTTTG, Nanog-R: AACCCACCCTTATAAATTCTCAATTA. PCR products were sequenced with the same primers, using BigDye Terminator v3.1 Cycle Sequencing Kit (Life Technologies, Paisley, UK), and an ABI3130XL automated sequencer (Life Technologies, Paisley, UK).

**Antibodies**

The following antibodies and dilutions were used in the course of this work: TRA-1-60, 1:100 (MAB4360, Millipore, Watford, UK), TRA-1-81, 1:100 (MAB4381, Millipore, Watford, UK), SSEA-4, 1:100 (MAB4304, Millipore, Watford, UK), Alpha smooth muscle actin 1:100 (ab5694, Abcam, Cambridge, UK), HN-IL 4.1 1:500 (DNAVEC, Tsukuba, Japan)**,** FOXA2 1:500 (ab40874, Abcam, Cambridge, UK) **,** GATA4 1:400 (AB4132, Millipore, Watford, UK). CD34 vioblue 1:10 (130-095-393, Miltenyi Biotec, Bisley, UK), Mouse IgG-2a Vioblue 1:10 (130-094-671, Miltenyi Biotec, Bisley, UK). H2AX, 1:100 (05-636, Millipore), Amyloid 6E10, (SIG-39320, Covance). Secondary antibodies were conjugated with Alexa Fluor (Life Technologies, Paisley, UK).

**Sendai virus expression**

RNA from iPSCs or SeV-transfected primary skin fibroblasts (as positive control), was isolated with RNeasy columns (Qiagen, Manchester, UK). cDNA was produced using superscript II reverse transcriptase (Life Technologies, Paisley, UK). PCR was performed as described above. Annealing temperature of 57°C for GAPDH, and 63°C for SEV. Primers: SEV-F: GGATCACTAGGTGATATCGAGC,

SEV-R: ACCAGACAAGAGTTTAAGAGATATGTATC,

GAPDH-F: ACCAGGGCTGCTTTTAACTC,

GAPDH-R: CATCGCCCCACTTGATTTTG.

Presence of SeV encoded protein was detected by immunofluorescence using an antibody against the SeV coat protein HN-IL 4.1.

**Teratomas**

3-4 million undifferentiated iPSCs (in DMEM-F12 with 10µM ROCK inhibitor Y-27632 (Tocris Bioscience) were injected subcutaneously per flank of 3 month old NOD-SCID mice. Teratomas were harvested approximately 2 months later, fixed for 24h in 4% v/v formalin in PBS, paraffin embedded sections were cut and stained with standard H&E.

**Genomic DNA characterization**

Confirmation of chromosome 21 copy number was achieved by testing two highly polymorphic and well characterized chromosome 21 STR loci (D21S11 and Penta D) contained within the PowerPlex 16 kit (Promega, Southampton, UK) for whole genome DNA fingerprinting for forensic purposes. Amplification of 1 ng DNA was performed in accordance with the manufacturer’s protocol, with products being separated on a ABI3130XL genetic analyzer (Life Technologies, Paisley, UK) and data analyzed with GeneMapper v3.2 software (Life Technologies, Paisley, UK). Confirmation of chromosome 21 copy number was independently verified by co-authors in Geneva, using 5 different HSA21 microsatelite markers. Array Comparative Genome Hybridisation (aCGH) and comparison was performed following the manufacturer’s conditions and protocol, using Agilent Human Genome CGH Microarray Kit, 4x44K (Ref # G4413A) designed for genome-wide DNA copy number variation profiling, containing 42,494 distinct biological features, with overall median probe spacing of 43 KB (24 KB in Refseq genes). Probes were annotated against NCBI Build 37 (UCSC hg19, February 2009). For data on integration methods reprogrammed iPSCs, the Supplementary Table S3 from Hussein et al.^2^ was filtered for size, by removing all events smaller than 44kbp. The remaining events were re-annotated according to NCBI Build 37 (UCSC hg19, February 2009), for comparison to our data. CNVs (excluding inversions) that vary commonly among healthy individuals in human populations were identified using Database of Genomic Variants (DGV) (http://projects.tcag.ca/variation/?source=hg19) and UCSC genome browser (hg19). All CNVs, or parts within CNVs that were not covered by DGV, and RefSeq genes within these intervals, were used for comparison to our aCGH data, which was filtered identically.

**iPSC differentiation**

Embryoid bodies (EBs) were formed after iPSC colonies were detached with dispase (1 mg/ml), for 20min, and by pipetting in EB medium (DMEM-F12 glutamax (Life Technologies, Paisley, UK), 10% (v/v) foetal calf serum, 1x Pen/Strep (Sigma, Dorset, UK), 1.4x non-essential amino acids (Sigma, Dorset, UK)). Colonies were transferred to a 15ml tube and left to settle by gravity. The medium was removed, and 5ml of medium was added, and the process was repeated. EB medium with 10µM ROCK inhibitor Y-27632 (Tocris Bioscience) (ROCK-i) (2ml/6-well) was added, and moved to an ultralow attachment plate (Corning). The next day, 1 ml EB medium with 10µM ROCK-i was added. Half the medium was exchanged daily, for 7 days.

Neuro EBs

Neuro EBs were generated using a spin EB protocol adapted from a published method^3^.

One day before generating NEBs, 70% confluent iPSCs were passaged to low density MEFs (1x10^5^/3.5 cm well). Following a short wash in PBS, iPSCs were treated with accutase (Sigma A6964) for 10 minutes. Dissociated cells were collected with a p1000 and washed twice in HESC media (1,200rpm, 5 minutes). Finally, cells were resuspended in 2ml HESC containing 10µM ROCK-inhibitor and plated onto MEFs. The following day, media was aspirated and cells were harvested with accutase, washed and resuspended in HESC with 10µM ROCK-inhibitor as above. Viable cells were counted using a Nucelocounter NC-200 and the appropriate number of cells was collected by centrifugation at 1,200 rpm, for 5 minutes. iPSCs were resuspended in NEB/PVA media (DMEM/F12 Glutamax (Invitrogen, 31331-028), supplemented with 0.4% w/v PVA (Sigma P8136), 1x penicillin/streptomycin (Sigma, P4333), 1 x N-2 Supplement (Invitrogen, 17502-048) and 1 x B-27 without vitamin A (Invitrogen, 12587-010)) with 10µM ROCK Inhibitor, and seeded at a density of 10,000 viable cells per well of a 96-well U-shaped non-treated plate (Corning Costar 3788). Plates were centrifuged at 480xg, for 3 minutes. The following day media was topped up 100 µl/well NEB/PVA, and half the volume of media was replaced every day from day 2-6. After 7 days NEBs were transferred to poly-ornithine/laminin coated 6-well plates using a wide bore p1000. Up to 10 NEBs were placed per well. NEBs were allowed to settle to the bottom of the well and media was changed to Neural Rosette media (DMEM/F12 Glutamax (Invitrogen, 31331-028), supplemented 1x penicillin/streptomycin (Sigma, P4333), 1 x N-2 Supplement (Invitrogen, 17502-048) and 1 x B-27 with vitamin A (Invitrogen, 17504-044)). Media was replaced every 2-3 days and rosettes were selected after 7 days using STEMDiff Neural Rosette Selection Reagent (Stem Cell Technologies, 05832) according to the manufacturer’s protocol. Selected rosettes were cultured as neural progenitor cells (NPCs) in poly-ornithine/laminin coated dishes, in NPC media (neural rosette media supplemented with 20ng/ml FGF-2). Media was changed every 2-3 days and NPCs were passaged as necessary using accutase. NPC cumulative population doublings were determined by seeding 0.5 x 10^6^ viable cells per 35mm well and counting the number of viable cells retrieved when next passaged.

Haemo EBs (HEBs)

HEBs were generated following a similar protocol as NEBs, but with the following adjustments. HEB media Stemdiff APEL medium (StemCell Technologies) supplemented with 40 ng/ml SCF, 20 ng/ml VEGF, and 20 ng/ml BMP4) was used in place of NEB media and only 3,000 cells were seeded per well. Two days after plating, 100µl HEB media was added to each well.

Haemo-EB Measurements

On day 5, images were acquired using the IN Cell 1000 high content microscope (GE) at 4x magnification, and analyzed using the Developer analysis software (Version 1.8, GE). No pre-processing of the original images was performed. For statistical analysis p values were determined using a two-tailed student’s T-test.

Neuronal differentiation via NeuroEBs

This was performed by a protocol modified from^4^. After 7 days, Neuro-EBs were transferred at a density of 10-20 per well of a 6 well plate well onto poly-ornithine/laminin treated plates in 1ml NeuroEB medium with 20 ng/ml FGF-2. The cells were incubated for 2 weeks. Medium was exchanged every second day. Neural progenitor cells (NPCs) were dissociated to a single cell suspension with accutase (5 minutes at 37°C). They were re-plated in poly-ornithine/laminin treated plates in neural-differentiation medium (Neurobasal medium (Life Technologies, Paisley, UK) 1xB27 (+ vitamin A) (Life Technologies, Paisley, UK), 10ng/ml BDNF (Peprotech, London, UK), Pen/Strep (Sigma, Dorset, UK), L-glutamine (Sigma, Dorset, UK)), at a density of 200,000/well of a 6-well plate. Medium was exchanged every other day.

Directed neuronal differentiation protocol was modified from published work^5-6^, iPSCs on MEFs were treated with accutase, washed twice with 5ml HESC, centrifuged for 5 minutes at 240xg. MEFs were removed by plating on gelatin coated dishes for 1 hour with HESC with FGF-2 (50 ng/ml)/ROCK-inhibitor (10µM). The non-adherent cells were plated on matrigel coated 12-well plates in TESR (Stemcell Technologies, Grenoble, France) with 8 ng/ml FGF-2 and ROCK-inhibitor (10µM). When 95% confluent neural induction was induced by changing the medium to 3N medium (1:1 mixture of N2 and B27) with 500 ng/ml Noggin-CF chimera (R&D systems, Abingdon, UK) and 10μm SB431542 (Tocris, Bristol, UK). 1 ml of medium was exchanged for 8-11 days (on d2, 3, 5, 7, 9 and 11). After 8-11 days, the medium was changed to 3N with 20 ng/ml FGF-2 for 2 days. The cells were lifted with accutase, washed twice, and 50,000 cells/cm^2^ were seeded on polyornithine/laminin treated plates (or cover slips). 3N medium with BDNF (Peprotech, London, UK) 20 ng/ml, was changed every other day. N2 medium: DMEM/F12 + glutamax (Life Technologies, Paisley, UK), 1x N2 (Life Technologies,Paisley, UK), 5 μg/ml insulin (Sigma, Dorset, UK), 1x non-essential amino acids (Sigma, Dorset, UK), 100 μM β-mercaptoethanol (100 μM),1x Pen/Strep (Sigma, Dorset, UK); B27 medium: Neurobasal medium (Life Technologies, Paisley, UK), 1x B27 (+ vitamin A) (Life Technologies, Paisley, UK), 2mM glutamine (Sigma, Dorset, UK), 1x Pen/Strep (Sigma, Dorset, UK).

**Electrophysiology**

Whole-cell patch clamp recording from disomic and trisomic cells was used to verify neuronal-like electrical behavior, as well as the presence of voltage-gated and ligand-gated ion channels. Cells were recorded either under voltage clamp, at a holding potential of -70 mV, or under current clamp at the resting membrane potential, using an Axopatch 200B amplifier (Molecular Devices, Wokingham, UK). Series resistance was compensated to at least 60%, and all signals were filtered at 5 kHz. Currents were digitized at 50 kHz using a Digidata 1322A (Molecular Devices, Wokingham, UK) and recorded to disk. Patch pipettes with a resistance of 3 - 4 MΩ, were filled with an intracellular solution containing (mM): 137 K-gluconate, 3 KCl_2_, 10 HEPES, 5 EGTA, 0.5 CaCl_2_, 2 MgCl_2_, 2 Na-ATP and 0.5 Na-GTP; pH 7.30 (adjusted with 1N KOH). Cells were continuously perfused with a Krebs solution containing (mM): 140 NaCl, 4.7 KCl, 1.2 MgCl_2_, 2.52 CaCl_2_, 11 Glucose and 5 HEPES; pH 7.4 (adjusted with 1M NaOH). To generate action potential bursts, a depolarising jump to -40 mV was delivered in current clamp. For ligand-gated currents under voltage clamp, 1 mM GABA or glycine solutions were applied for 5 s to cells using a U-tube application system^7^.

**Live fluorescent imaging of calcium transients**

The protocol was adapted from^8^. Briefly, Oregon Green 488 BAPTA-1 AM, (Molecular Probes UK, #O6807) was reconstituted in DMSO and plurionic acid F-127 solution (Molecular Probes UK, #P3000MP), then further diluted in culture medium to a final concentration of 3.5μg/ml. After 6 weeks of differentiation following the directed differentiation protocol, iPSC-derived neurons were incubated with the dye for 30-40 minutes, medium was replaced and cells were incubated for a further 20 minutes before imaging.

Cells were imaged by live cell microscopy on an inverted epi-fluorescence microscope.

Images were captured at 20 frames per second for 1 minute (1,200 frames). Image analysis, segmentation and thresholding was carried out using MetaMorph software.

**Mitochondrial live staining**

Live neurons were incubated with 10µM JC-10 (Enzo Life Sciences, Exeter, UK) and Hoechst for 30 minutes, then washed three times with warm media before imaging on the ImageXpress Micro XL System (Molecular Devices) as described below.

**Immunostaining**

Cell fixation was carried out using 4% paraformaldehyde. For amyloid labelling, neurons were permeabilised in 0.1% NP40 in PBS for 20 minutes then blocked in 10% FCS in PBS with 0.5% Tween20 (PBST) for 1 hour. Primary antibody was diluted in PBST and incubated on cells overnight at 4°C. Secondary antibody was diluted in PBST and incubated on cells for 2 hours. Hoechst (Hoechst 33342, Life Technologies #R37605) was included in secondary antibody incubation. PBST was used for all washes.

For γH2AX labelling, neurons were permeabilised in 0.1% TritonX-100 in PBS for 10 minutes then blocked in 0.25% BSA in PBS for 1 hour. Primary antibody was diluted in blocking buffer and incubated on cells for 2 hours. Secondary antibody was also diluted in blocking buffer and incubated on cells for 1 hour. Hoechst was included in secondary antibody incubation. PBS was used for all washes.

**High Content microscopy imaging and multiparametric image Analysis**

Image capture and quantification was performed using automated multi-parametric analysis on the ImageXpress Micro XL (Molecular Devices) wide-field high content imaging system, and data analysed using MetaMorph (Version 4.0.0.43) software. All neuronal images were taken using the 40x objective and a minimum of 25 fields were imaged per well.

For amyloid quantification the integrated intensity of the fluorescent signal was calculated using the multiwavelength analysis function of MetaMorph. Three wells per cell line and a minimum of 6,000 cells per well were analyzed.

For H2AX, the number of foci in the nuclei were calculated using the granule analysis function of MetaMorph (Version 4.0.0.43) software. Three wells per cell line and a minimum of 6,000 cells per well were analyzed.

Mitochondrial size and number were quantified after live staining with JC-10 using the granule analysis function of MetaMorph (Version 4.0.0.43) software. Green JC-10 signal (reduced mitochondrial membrane potential) was analyzed using multiwavelength analysis. A total of 4 wells and a minimum of 1,500 cells per cell line were imaged and analyzed.

**Supplementary References**

1. Ban H, Nishishita N, Fusaki N, et al. Efficient generation of transgene-free human induced pluripotent stem cells (iPSCs) by temperature-sensitive Sendai virus vectors. *Proc Natl Acad Sci U S A.* 2011;108:14234-14239.
2. Hussein SM, Batada NN, Vuoristo S, et al. Copy number variation and selection during reprogramming to pluripotency. *Nature.* 2011;471:58-62.
3. Ng ES, Davis RP, Hatzistavrou T et al. Directed differentiation of human embryonic stem cells as spin embryoid bodies and a description of the hematopoietic blast colony forming assay. *Current protocols in stem cell biology*. Jan 2008;Chapter 1:Unit 1D 3
4. Brennand KJ, Simone A, Jou J, et al. Modelling schizophrenia using human induced pluripotent stem cells. *Nature.* 2011;473:221-225.
5. Chambers SM, Fasano CA, Papapetrou EP, et al. Highly efficient neural conversion of human ES and iPS cells by dual inhibition of SMAD signaling. *Nat Biotechnol.* 2009;27:275-280.
6. Shi Y, Kirwan P, Livesey FJ. Directed differentiation of human pluripotent stem cells to cerebral cortex neurons and neural networks. *Nat Protoc.* 2012;7:1836-1846.
7. Mortensen M, Smart TG. Single-channel recording of ligand-gated ion channels. *Nat Protoc.* 2007;2:2826-2841.
8. Trevelyan AJ, Kirby DM, Smulders-Srinivasan TK et al. Mitochondrial DNA mutations affect calcium handling in differentiated neurons. *Brain.* 2010;133: 787-796.
